# Effect of Acetic Acid Concentration on Pore Structure for Mesoporous Bioactive Glass during Spray Pyrolysis

**DOI:** 10.3390/ma11060963

**Published:** 2018-06-06

**Authors:** Bo-Jiang Hong, Chih-Wei Hsiao, Fufa Fetene Bakare, Jung-Ting Sun, Shao-Ju Shih

**Affiliations:** Department of Materials Science and Engineering, National Taiwan University of Science and Technology, Taipei 10607, Taiwan; M10404315@mail.ntust.edu.tw (B.-J.H.); martin811024@gmail.com (C.-W.H.); D10504825@mail.ntust.edu.tw (F.F.B.); M10504320@mail.ntust.edu.tw (J.-T.S.)

**Keywords:** mesoporous bioactive glass, spray pyrolysis, acetic acid

## Abstract

Mesoporous bioactive glass (MBG) is considered as one of the most important materials in the field of bone implants and drug carriers, owing to its superior bioactivity. In previous studies, tri-block surfactants (e.g., F127 and P123) were commonly used as pore-forming agents. However, the use of surfactants may cause serious problems such as micelle aggregation and carbon contamination and thus decrease bioactivity. Therefore, in this study, we demonstrated the synthesis of MBG using acetic acid (HAc) as a pore-forming agent to overcome the disadvantages caused by surfactants. Both untreated and HAc-treated BG powders were synthesized using spray pyrolysis and various characterizations were carried out. The results show that a mesoporous structure was successfully formed and the highest specific surface area of ~230 m^2^/g with improved bioactivity was reported.

## 1. Introduction

Bioactive glass (BG) is a non-toxic, biocompatible, and bioactive material, developed in the 1960s [1,2]. Among these properties, the bioactivity has attracted much attention and been investigated extensively to enhance BG’s performance [3]. It is well known that BG has the capability to form hydroxyl apatite (HA) layers once immersed in simulated body fluid (SBF), and the formation of HA is influenced by factors such as compositions [4,5] and surface areas [6]. The composition factor has been studied by Li et al. [7], who demonstrated that the composition of 57S presents the best bioactivity. In addition, the factor of surface areas was first reported by Yan et al. in 2004 [8]. By creating a mesoporous structure within BG particles, they increase the surface areas and hence increase bioactivity.

To synthesize BG, several studies used the sol-gel method owing to its chemical flexibility [8]. However, the whole process takes a few days and it is a batch production. In contrast, the spray pyrolysis (SP) process offers the benefits of low cost, short process time, and continuous fabrication, which is suitable for mass production [9,10]. In addition, to fabricate mesoporous bioactive glass (MBG), surfactants such as F127 (EO_100_PO_65_EO_100_) [11] and P123 (EO_20_PO_70_EO_20_) [12] were commonly used as pore-forming agents, where EO is poly ethylene oxide and PO is poly propylene oxide. However, the use of surfactants may cause problems such as micelle aggregation and carbon contamination. The micelle aggregation will produce particles with a wrinkled surface and hence reduce the porosity and surface area of the resulting specimen [13]. In addition, carbon contamination will inhibit the formation of HA. Both phenomena will result in decrease of bioactivity and hence there is a need to develop a surfactant-free process to synthesize MBG.

Therefore, we proposed to use acetic acid (HAc) as the new pore-forming agent owing to its unique decomposition behavior, CH_3_COOH + 2O_2_ → 2CO_2_ + 2H_2_O. It generates large amounts of CO_2_ and H_2_O gases, which create the pore structure during calcination [14]. Moreover, the decomposition temperature of HAc (440 °C) [15] is much lower than the calcination temperature of BG (550 °C) [16], which allows the decomposition reaction of HAc to be complete. Hence, HAc can replace the surfactants as the new pore-forming agents for two reasons: (i) It has lower molecular weight (60 g/mol molecular weight [17]) as compared to F127 (12,600 g/mol molecular weight [18]) and P123 (5750 g/mol molecular weight [18]), which minimizes carbon contamination; (ii) since HAc is completely soluble in water, no aggregation effect needs to be considered.

In this study, untreated and three HAc-treated BG powders were prepared using SP. Characterizations of phase composition, surface morphology, and inner morphology of all BG powders were obtained using X-ray diffraction (XRD), scanning electron microscopy (SEM), and transmission electron microscopy (TEM), respectively. The detailed pore sizes, pore volumes and surface areas were examined using nitrogen adsorption/desorption isotherms along with Brunauer-Emmett-Teller (BET) [19] and Dollimore–Heal (DH) method [20]. At last, the bioactive tests were carried out and characterized using a Fourier transform infrared reflection (FTIR) spectrophotometer.

## 2. Materials and Methods

### 2.1. Synthesis

In this study, both untreated (solid) and HAc-treated (mesoporous) BG powders based on 57S composition (57 mol % SiO_2_, 33 mol % CaO, and 10 mol % P_2_O_5_) were prepared using tetraethyl orthosilicate (TEOS, Si(OC_2_H_5_)_4_, 99.9 wt %, Showa, Tokyo, Japan), calcium nitrate tetrahydrate (CN, Ca(NO_3_)_2_·4H_2_O, 98.5 wt %, Showa, Tokyo, Japan), and triethyl phosphate (TEP, (C_2_H_5_)_3_PO_4_, 99.0 wt %, Alfa Aesar, Heysham, UK) as the sources of Si, Ca and P, respectively. The precursor solution of untreated BG was prepared by dissolving 37.5 g TEOS, 24.78 g CN and 0.73 g TEP in 1.60 g of 0.5 M HCl and 60.00 g of ethanol. As for HAc-treated BG, an additional pore-forming agent of HAc (99.8 wt %, Honeywell, NJ, USA) with various concentrations of 1M, 2M and 3M were added into the precursor solutions. All precursor solutions for untreated and HAc-treated BGs were stirred at room temperature for 4 h for homogeneity before the SP process.

For the SP process, all precursor solutions (the mixture of the 200 mL precursor solution and 1000 mL DI-water) were dispersed into fine droplets at the frequency of 1.65 MHz using an ultrasonic nebulizer (KT-100A, King Ultrasonic, New Taipei, Taiwan) with the applied pressure of 1.013 × 10^5^ Pa. With the air carrier gas, the droplets were led into a tube furnace (D-110, Dengyng, New Taipei, Taiwan) with three heating zones of 200 °C, 550 °C, and 300 °C, which underwent preheating, calcining, and cooling to form BG particles [21]. The surfaces of the resulting particles were charged by electrons released from tungsten corona wire at high voltage of 16 kV. Finally, the negatively charged particles were neutralized and condensed inside an earthed stainless steel collector.

### 2.2. Characterization

Characterizations of the phase composition were obtained using a XRD diffractometer (D2 Phaser, Bruker, Karlsruhe, Germany) with collection angles ranging from 20° to 80°. Surface and inner morphologies were examined using a field-emission scanning electron microscope (SEM, JSM-6500F, JEOL, Tokyo, Japan) and a field-emission transmission electron microscope (TEM, Tecnai G2 F20, FEI, Hillsoboro, OR, USA), respectively. The particle size distributions were measured with more than 300 particles from a couple of SEM images to ensure its reliability. In addition, a constant-volume adsorption apparatus (Novatouch LX2, Quantachrome Instruments, Boynton Beach, FL, USA) was operated at −196 °C to obtain nitrogen adsorption and desorption isotherms. The specific surface areas, pore volumes, and pore sizes of all BG powders were computed using BET method [19] and DH method [20].

Bioactivity tests of all BG powders were carried out using SBF, which has ionic concentration similar to human plasma [22]. The specimens were prepared by immersing the powders in SBF with different solid-to-liquid ratios: 2 mg to 10 mL for untreated BG powder, 2 mg to 42 mL for 1M HAc-treated BG powder, 2 mg to 59 mL for 2M HAc-treated BG powder, and 2 mg to 51 mL for 3M HAc-treated BG powder. Each specimen was immersed at 37 °C for 1 h and examined using FTIR once dry.

## 3. Results

### 3.1. Crystallographic Structure and Morphology

**Figure 1:**
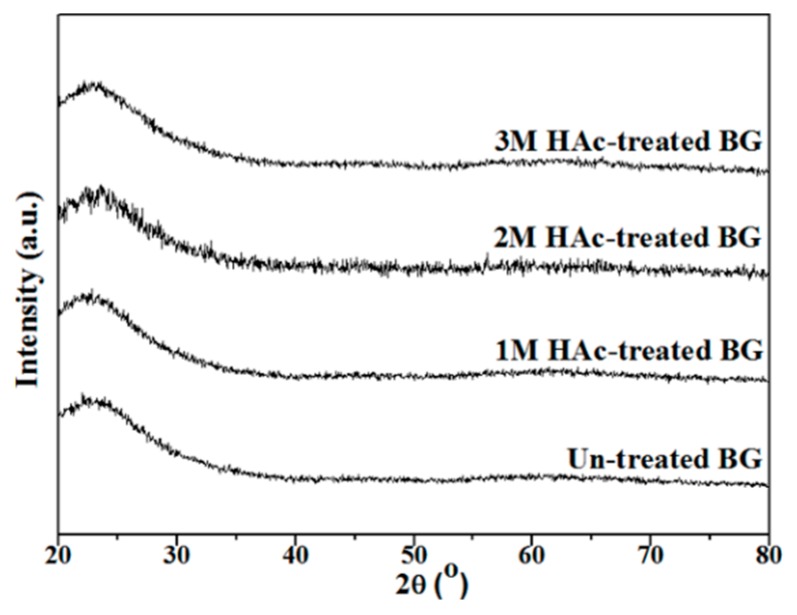
XRD patterns of un-treated and HAc-treated BG powders.

Figure 1 shows the XRD patterns of un-treated and HAc-treated BG powders. A broad band between 20° to 40° can be observed from all patterns with absence of the crystalline phase, which indicates that all BG powders exhibited amorphous phase after the SP process.

**Figure 2:**
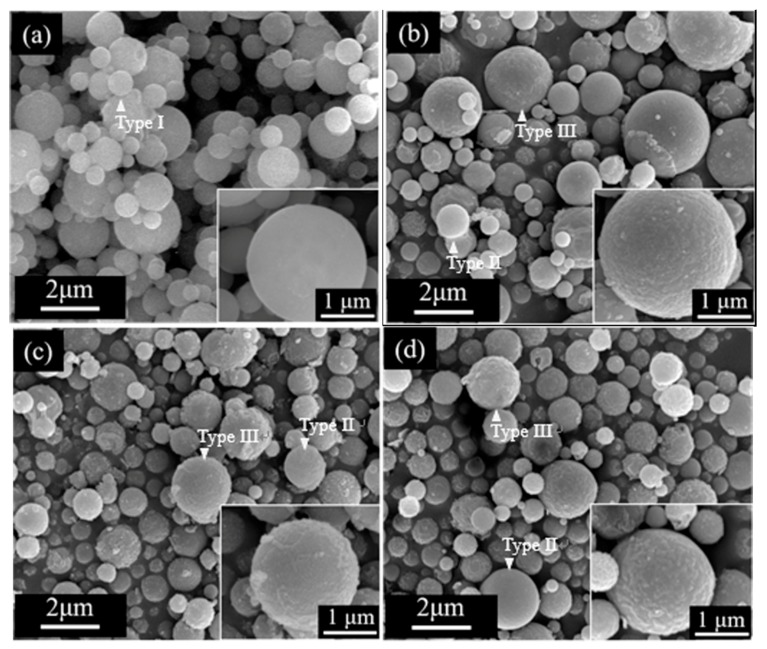
SEM images of (**a**) untreated BG powders, and (**b**) 1M, (**c**) 2M, and (**d**) 3M HAc-treated BG powders. The insets are the enlarged particles for corresponding morphologies.

Figure 2 shows the SEM images of all BG powders and typical spherical particles from the SP process can be observed among all images. In addition, it can be seen from Figure 2a that the untreated BG powder presents only one surface morphology of smooth sphere. In contrast, two surface morphologies of smooth and concave sphere (see insets) can be found in the HAc-treated BG powder as shown in Figure 2b–d.

**Figure 3:**
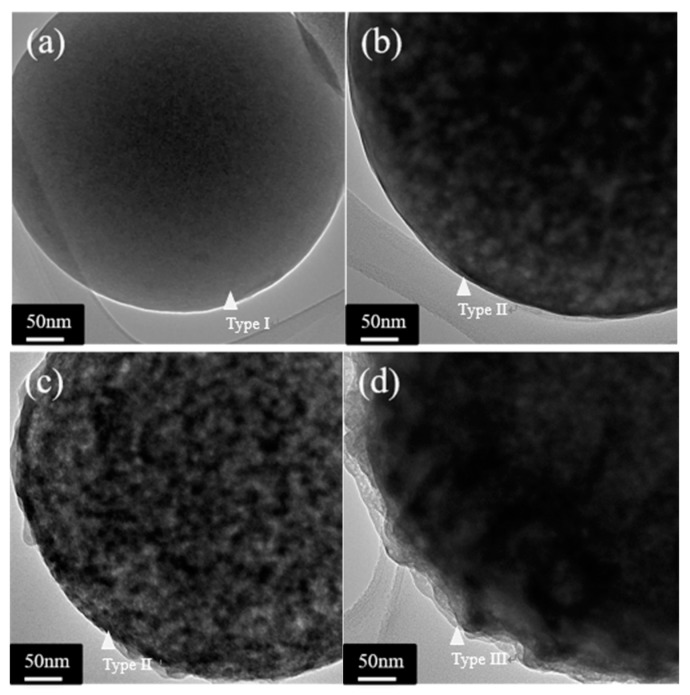
TEM micrographs of (**a**) untreated BG powders, and (**b**) 1M, (**c**) 2M, (**d**) 3M HAc-treated BG powders.

Figure 3 shows the TEM micrographs of all BG particles and it can be seen from Figure 3a that the untreated BG particle presents a no-contrast within the particle. This indicates there are no thickness variations and thus represents its inner morphology as solid. In addition, contrast of intensities can be found in HAc-treated BG particles as shown in Figure 3b–d. The dark regions represent higher absorption of electrons, while bright regions represent with lower absorption of electrons, thus indicating the porous morphology of the HAc-treated BG particles. Combining both SEM and TEM results, the morphologies of all BG particles can be categorized into three types: the untreated BG particles exhibits the morphology of solid smooth sphere (Type I), while the HAc-treated BG particles have two morphologies of porous smooth sphere (Type II) and porous concave sphere (Type III). In addition, the surface of type III has a higher roughness than the surface of type II. The surface roughness may correlates with pore sizes of the internal microstructure.

**Figure 4:**
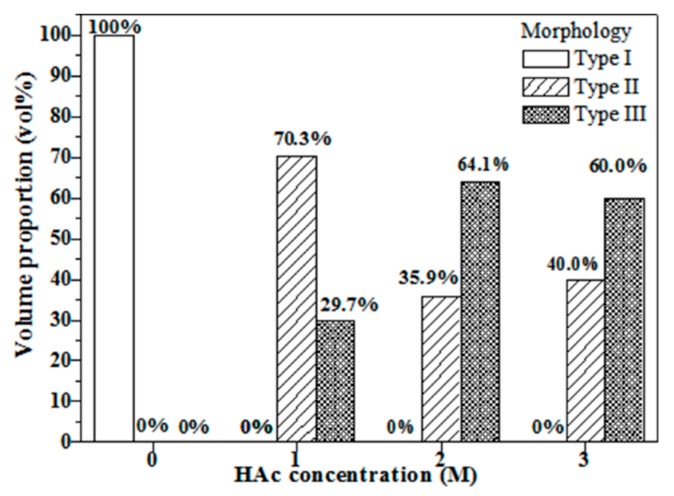
Statistical measurements of morphology types of untreated and HAc-treated BG powders.

The statistical measurement of particle morphology types from SEM images is shown in Figure 4. The result shows that the untreated BG powder contains only Type I particles, whereas all HAc-treated BG powders associate with only Type II and III particles. It can also be seen that with an increase of HAc concentration from 1M to 2M, the proportion of Type III porous concaved particles increases from 29.7% to 64.1%; the proportion decreases slightly to 60.0% for HAc concentration of 3M.

### 3.2. Particle Sizes and Specific Surface Areas

The average particle sizes and standard deviations of the untreated and 1M, 2M, 3M HAc-treated BG powders are 842 ± 469, 947 ± 269, 800 ± 183, 1010 ± 280 nm, respectively; all particles are in the same size range.

**Figure 5:**
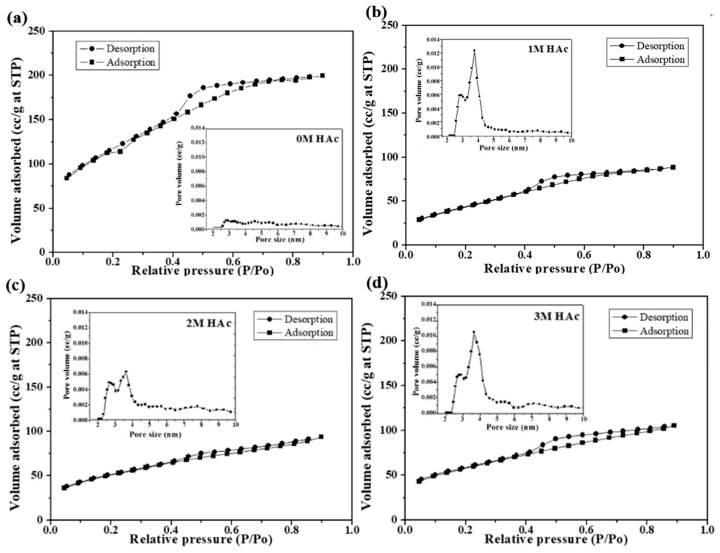
Nitrogen adsorption and desorption isotherms of (**a**) untreated BG powders, and (**b**) 1M, (**c**) 2M, and (**d**) 3M HAc-treated BG powders. The inserts are their corresponding pore size distributions.

**Figure 6:**
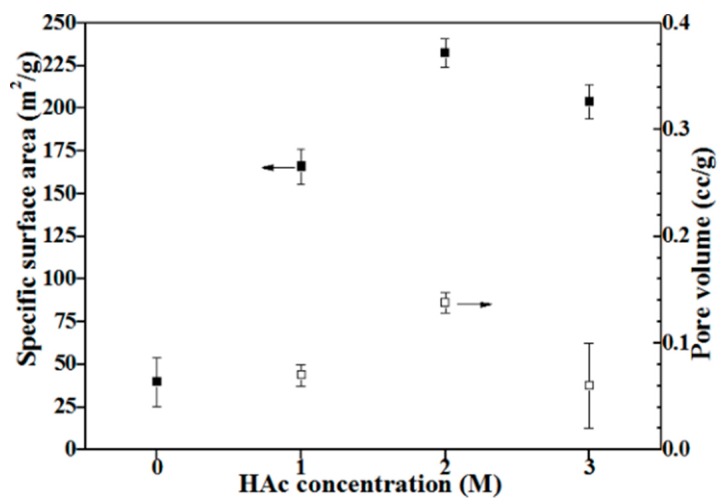
Specific surface areas and pore volumes of untreated and HAc-treated BG powders.

Figure 5 shows nitrogen adsorption and desorption isotherms with insets of pore size distributions of all BG powders. Based on the original IUPAC classification, all BET isotherms are identified as H4 loops, which indicates that all BG powders contain both open and partially blocked mesopores [23]. From the pore size distribution profile, it is evident that the HAc-treated BG powders exhibit a mesoporous structure with pore sizes ranging from 2–5 nm. Moreover, specific surface areas and pore volumes are computed using BET methods and shown in Figure 6. It can be seen from Figure 6 that untreated BG powder has the lowest surface area of 39.7 m^2^/g, while the value increases significantly to more than 160 m^2^/g with the treatment of HAc and reaches the maximum of 232.7 m^2^/g of 2M HAc-treated BG powder. In addition, the resulting pore volumes show the same trend as surface area with range from 0.07 to 0.138 cc/g.

### 3.3. In Vitro Bioactivity

**Figure 7:**
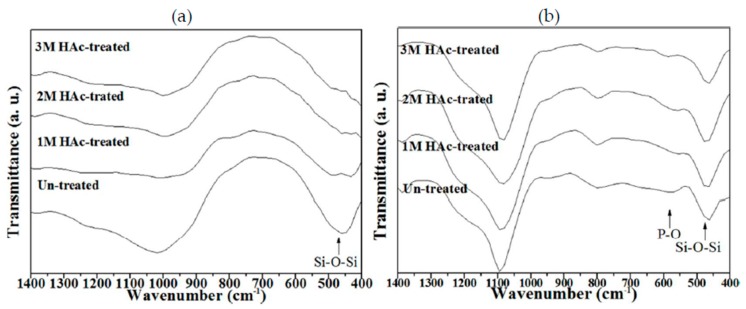
FTIR spectra of untreated and HAc-treated BG powders (**a**) before and (**b**) after immersing in SBF for 6 h.

Figure 7 shows the FTIR spectra for the bioactivity tests. The bioactivities were determined by the deviation of the peak intensities, I_1_/I_2_, from the FTIR spectra [16,24], where I_1_ refers to the intensity of the P-O bending vibration around 566 cm^−1^ [11], and I_2_ refers to the intensity of the Si-O-Si bending vibration at 482 cm^−1^ [25]. Initially, for the as-prepared BG powders (Figure 7a), the FTIR spectra show a clear I_2_, but without any I_1_. These spectra suggest that these BG surfaces exhibit only Si-O-Si structure, but no P-O structure. Therefore, this result suggests no existence of HA on the BG surfaces before immersing in SBF. For the immersed BG powders, following Figure 7b, the spectra show the existence of I_1_ and I_2_, and the resulting I_1_/I_2_ values of untreated and 1M, 2M, 3M HAc-treated BG powders were 0.15, 0.35, 0.61, and 0.55, respectively. This indicates that BG powder treated with 2M HAc has the best bioactivity.

## 4. Discussion

Combining SEM and TEM results given in Figure 2 and Figure 3, particle morphology can be categorized into three types (Figure 4 shows their statistical measurements). The formation of Type I particles follows typical volume precipitation and has been well documented in our previous work [26]. Moreover, the porous structure of Type II and Type III particles is formed during the decomposition of the HAc. Following CH_3_COOH + 2O_2_ → 2CO_2_ + 2H_2_O, H_2_O and CO_2_ were formed, while HAc decomposed in the tube furnace at 440 °C [14,27]. The resulting CO_2_ gas then releases from the droplets, thereby creating a porous structure within the particles. Furthermore, the possible formation mechanism of Type III concave surface is given below. The interaction between HAc and TEOS in the precursor solutions might form into ethyl acetate [28]; these products will aggregate on the surface of the particles and form the concave surface after calcination [29]. The higher HAc concentration induces a larger amount of ethyl acetate, and thus results in a higher proportion of Type III particles.

**Figure 8:**
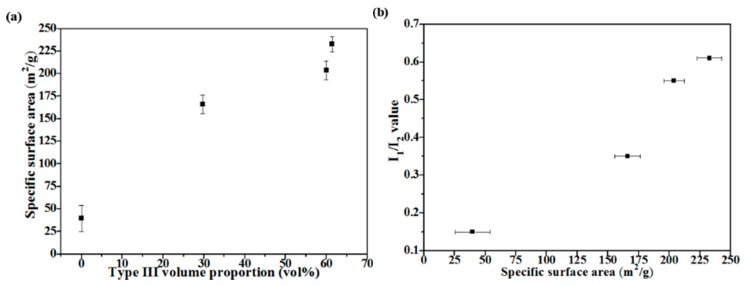
(**a**) Correlations between specific surface area and Type III particles and (**b**) bioactivity and specific surface area of all BG powders.

Figure 8 shows the correlation of particle morphology, specific surface area, and bioactivity. Figure 8a clearly shows that the specific surface area increases when the volume proportion of Type III increases. This implies that the concave surface is enhancing the specific surface area of BG particles. Next, Figure 8b shows the comparison between specific surface areas and bioactivity of all BG powders. The graph shows that BG powders with higher specific surface area correspond to the higher value of I_1_/I_2_ (i.e., greater HA formation), which agree well with previous results on MBG studies [13,30,31]. Finally, to the best of our knowledge, the surface area achieved in this study of 232.7 m^2^/g exceeds the P123-treated MBG particles of 165.0 m^2^/g [32] and thus becomes the highest specific surface area reported for SP-derived MBG powders to date.

## 5. Conclusions

In this study, both untreated and HAc-treated BG powders were successfully synthesized using SP. The results show that a mesoporous structure is formed with HAc-treated BG powders and surface area is increased up to 230 m^2^/g. Bioactive tests were carried out, confirming increased bioactivity. Therefore, HAc has demonstrated its potential to be used as a new pore forming agent for future studies.

## References

[1] Hench L.L., Splinter R.J., Allen W.C., Greenlee T.K. (1971). Bonding mechanisms at the interface of ceramic prosthetic materials. J. Biomed. Mater. Res..

[2] Rahaman M.N., Day D.E., Bal B.S., Fu Q., Jung S.B., Bonewald L.F., Tomsia A.P. (2011). Bioactive glass in tissue engineering. Acta Biomater..

[3] Lei B., Chen X.F., Wang Y.J., Zhao N.R., Du C., Fang L.M. (2009). Synthesis and in vitro bioactivity of novel mesoporous hollow bioactive glass microspheres. Mater. Lett..

[4] Hench L.L. (2006). The story of bioglass. J. Mater. Sci. Mater. Med..

[5] Xia W., Chang J. (2008). Preparation, in vitro bioactivity and drug release property of well-ordered mesoporous 58S bioactive glass. J. Non-Cryst. Solids.

[6] Mačković M., Hoppe A., Detsch R., Mohn D., Stark W., Spiecker E., Boccaccini A. (2012). Bioactive glass (type 45S5) nanoparticles: In vitro reactivity on nanoscale and biocompatibility. J. Nanopart. Res..

[7] Li R., Clark A., Hench L.L. (1991). An investigation of bioactive glass powders by sol-gel processing. J. Appl. Biomater..

[8] Yan X., Yu C., Zhou X., Tang J., Zhao D. (2004). Highly ordered mesoporous bioactive glasses with superior in vitro bone-forming bioactivities. Angew. Chem. Int. Ed..

[9] Shih S.-J., Wu Y.-Y., Chen C.-Y., Yu C.-Y. (2012). Morphology and formation mechanism of ceria nanoparticles by spray pyrolysis. J. Nanopart. Res..

[10] Chou Y.J., Hong B.J., Lin Y.C., Wang C.Y., Shih S.J. (2017). The correlation of pore size and bioactivity of spray-pyrolyzed mesoporous bioactive glasses. Materials.

[11] Lei B., Chen X., Wang Y., Zhao N., Du C., Fang L. (2009). Synthesis and bioactive properties of macroporous nanoscale SiO_2_–CaO–P_2_O_5_ bioactive glass. J. Non-Cryst. Solids.

[12] Vallet-Regí M. (2001). Ceramics for medical applications. J. Chem. Soc. Dalton Trans..

[13] Shih S.-J., Lin Y.-C., Panjaitan L.V.P., Sari D.R.M. (2016). The Correlation of Surfactant Concentrations on the Properties of Mesoporous Bioactive Glass. Materials.

[14] Bush A., Gabriel R. (1985). The lungs in uraemia: A review. J. R. Soc. Med..

[15] Siahpoosh S.M., Salahi E., Hessari F.A., Mobasherpour I. (2017). Facile synthesis of γ-alumina nanoparticles via the sol-gel method in presence of various solvents. Sigma J. Eng. Natl. Sci..

[16] Shih C., Chen H., Huang L., Lu P., Chang H., Chang I. (2010). Synthesis and in vitro bioactivity of mesoporous bioactive glass scaffolds. Mater. Sci. Eng..

[17] López-Delgado A., Cano E., Bastidas J., López F. (1998). A laboratory study of the effect of acetic acid vapor on atmospheric copper corrosion. J. Electrochem. Soc..

[18] Alexandridis P., Hatton T.A. (1995). Poly (ethylene oxide)-poly (propylene oxide)-poly (ethylene oxide) block copolymer surfactants in aqueous solutions and at interfaces: Thermodynamics, structure, dynamics, and modeling. Colloids Surf. A Physicochem. Eng. Aspects.

[19] Brunauer S., Emmett P.H., Teller E. (1938). Adsorption of Gases in Multimolecular Layers. J. Am. Chem. Soc..

[20] Dollimore D., Heal G. (1964). An improved method for the calculation of pore size distribution from adsorption data. J. Chem. Technol. Biotechnol..

[21] Shih S.-J., Chou Y.-J., Panjaitan L.V.P. (2013). Synthesis and characterization of spray pyrolyzed mesoporous bioactive glass. Ceram. Int..

[22] Kokubo T., Kushitani H., Sakka S., Kitsugi T., Yamamuro T. (1990). Solutions able to reproduce in vivo surface-structure changes in bioactive glass-ceramic A-W. J. Biomed. Mater. Res..

[23] Thommes M., Kaneko K., Neimark A.V., Olivier J.P., Rodriguez-Reinoso F., Rouquerol J., Sing K.S. (2015). Physisorption of gases, with special reference to the evaluation of surface area and pore size distribution (IUPAC Technical Report). Pure Appl. Chem..

[24] Hench L.L. (1991). Bioceramics: From concept to clinic. J. Am. Ceram. Soc..

[25] Kay M.I., Young R., Posner A. (1964). Crystal structure of hydroxyapatite. Nature.

[26] Shih S.-J., Chou Y.-J., Chen C.Y., Lin C.-K. (2014). One-step synthesis and characterization of nanosized bioactive glass. J. Med. Biol. Eng..

[27] Djelloul A., Bouzid K., Guerrab F. (2008). Role of substrate temperature on the structural and morphological properties of ZnO thin films deposited by ultrasonic spray pyrolysis. Turk. J. Phys..

[28] Arenas L.T., Simm C.W., Gushikem Y., Dias S.L., Moro C.C., Costa T.M., Benvenutti E.V. (2007). Synthesis of silica xerogels with high surface area using acetic acid as catalyst. J. Braz. Chem. Soc..

[29] Shih S.-J., Tzeng W.-L. (2014). Manipulation of morphology of strontium titanate particles by spray pyrolysis. Powder Technol..

[30] Yan X., Huang X., Yu C., Deng H., Wang Y., Zhang Z., Qiao S., Lu G., Zhao D. (2006). The in-vitro bioactivity of mesoporous bioactive glasses. Biomaterials.

[31] Shih S.-J., Chou Y.-J., Borisenko K. (2013). Preparation method: Structure–bioactivity correlation in mesoporous bioactive glass. J. Nanopart. Res..

[32] Shih S.J., Sari D.R.M., Lin Y.C. (2016). Influence of chemical composition on the bioactivity of spray pyrolyzed mesoporous bioactive glass. Int. J. Appl. Ceram. Technol..

